# Migration, Habitat and Hunting Style Do Not Affect the Malar Stripe of Different Falcon Species

**DOI:** 10.1002/ece3.71028

**Published:** 2025-04-27

**Authors:** Celeste Polak, Jente Ottenburghs

**Affiliations:** ^1^ Wildlife Ecology and Conservation Wageningen University Wageningen the Netherlands; ^2^ Forest Ecology and Forest Management Wageningen University Wageningen the Netherlands

**Keywords:** adaptation, foraging ecology, morphological traits, solar glare hypothesis, species distribution

## Abstract

The solar glare hypothesis suggests that the malar stripe of a falcon decreases the sun's glare in the eye, possibly increasing their hunting success. The amount of sunlight an individual experiences could be affected by its migratory strategy, hunting style or main habitat. However, it is not known if these environmental variables impact the size and intensity of the malar stripe. Therefore, this study aimed to analyse differences in size and intensity of malar stripes between individuals of 12 falcon species with different migratory strategies, habitats, and hunting styles distributed worldwide. The malar stripes of 12 falcon species were measured and scored using more than 6000 photos from citizen science repositories. The measurements of the malar stripes were first reduced with a principal component analysis (PCA) and then analysed using a linear mixed model that included migratory strategy, habitat and hunting style as fixed factors and several posture variables as random factors. The relationships between the measurements of the malar stripe and solar radiation of the individual's location were also analyzed using linear mixed models. Overall, we found no differences in malar stripe size and intensity between species with differing migratory strategies, habitats, and hunting styles. The relationships between various characteristics of the malar stripe and solar radiation did depend on the species and the habitat the individual occupied. Therefore, migratory strategy, habitat and hunting style do not markedly influence the size and intensity of malar stripes across falcon species, suggesting that other mechanisms, such as thermoregulation or camouflage, also play a role.

## Graphical abstract

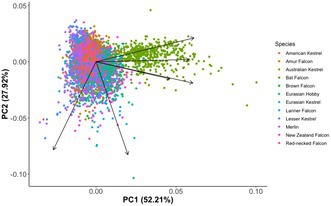

## 1 Introduction

Animal colour patterns can have various functions, such as camouflage, physiology, and communication (Burtt 1986; Caro 2005; Ortolani 1999). Multiple studies have shown that the colour patterns of animals can be adapted to the environment in which the animal lives (Galeotti et al. 2003; Marcondes et al. 2021; Marcondes et al. 2020; Romano et al. 2019; Roulin and Randin 2015; Tate et al. 2016; Théry 2006). For example, Delhey (2018) found darker species of Australian landbirds in humid areas and lighter species in warmer regions. Intraspecific adaptations to different environments have also been found, such as the plumage patterns of the Little Greenbul ( *Andropadus virens* ) that differ between habitats (Smith et al. 2008) or the colour differences in two subspecies of the Prairie Warbler ( *Dendroica discolor* ), with distinct migratory strategies (Buerkle 2000). These examples suggest that adaptations to the environment can result in regional phenotypic variation between individuals of the same species (Tate et al. 2016; Zink and Remsen 1986).

Dark plumage patches below the eye (hereafter: malar stripe) are an example of colour patterns that might be an adaptation to an animal's environment. These markings are mostly found on animal species that live and hunt in bright environments (Burtt 1986; Ficken et al. 1971). The solar glare hypothesis states that the malar stripe reduces the amount of sunlight that reflects in the eyes of the animal (Ficken and Wilmot 1968; Lebow 2020; Vrettos et al. 2021). Sunlight could affect an animal's ability to detect visual information (Martin and Katzir 2000), making it harder for predators to detect prey. Because of the malar stripe, the individuals might see objects, and thus prey, more clearly, which could increase their foraging success (Burtt Jr. 1984; Yosef et al. 2012). This phenomenon has been observed throughout multiple taxa, including birds (Burtt 1986; Caro 2009; Ortolani 1999; Santana et al. 2012), and even athletes are known to apply black paint under their eyes to improve their vision (DeBroff and Pahk 2003; Powers 2005).

In birds, there is indirect evidence that malar stripes are an adaptation to solar radiation. Vrettos et al. (2021) and Vrettos (2023) studied the effect of solar radiation on the size and colour intensity of the malar stripe of falcon species. Vrettos et al. (2021) found strong support for the solar glare hypothesis in Peregrine Falcons ( *Falco peregrinus* ), showing that the size and intensity of malar stripes of Peregrine Falcons are positively associated with average solar radiation, but were not influenced by other environmental factors, such as temperature or rainfall. However, Vrettos (2023) found no universal support for the solar glare hypothesis in other falcon species besides the Peregrine Falcon. The 39 falcon species she included in her study were distributed worldwide, inhabited different habitats, had different migratory strategies, and used different hunting styles. However, apart from habitat openness, these factors were not directly considered in the analyses.

Migration might complicate the relationship between solar radiation and the size and intensity of the malar stripe. Migratory species move from their wintering grounds to their breeding grounds and vice versa (Dingle and Drake 2007); sedentary species stay in the same area year‐round, and in partially migratory species, some individuals migrate and others remain sedentary (Buchan et al. 2020; García‐Silveira et al. 2022; Hoffman and Collopy 1988; Hoffman et al. 2002; Holte et al. 2016; Jahn et al. 2004). On average, migrating individuals could experience more sunlight than sedentary individuals because they move from areas with decreasing day length to regions with more daylight hours.

In their analysis of the Peregrine Falcon, a partially migratory species (White et al. 2020), Vrettos et al. (2021) only accounted for migration by performing two repeat analyses; one included only photos of non‐migratory subspecies and photos that were taken in the breeding season in the northern hemisphere (March–August), and the other included all photos except the ones taken in the USA and Canada. In her study of 39 falcon species, Vrettos (2023) excluded passage migrants from her analysis and only included solar radiation data for the 6 month breeding period for migratory species. Thus, migration was not directly considered in the analyses of both Vrettos et al. (2021) and Vrettos (2023).

In addition, habitat can affect the amount of solar radiation an individual experiences. Differences in vegetation density cause differences in light intensity between habitats (Endler 1993). For example, solar radiation in a forest is lower than in an open grassland (Endler 1993). Falcon species inhabit different types of habitats, such as grassland, shrubland, woodland, and forests, with varying densities of vegetation and, thus, different light intensities (Tobias et al. 2022). Hence, according to the solar glare hypothesis, individual falcons living in various habitat types might show differences in the size and intensity of their malar stripes.

The hunting style of the individual could also affect the amount of sunlight an individual experiences throughout the day (Elkins 2010). Following Tobias et al. (2022), we discriminate between five different hunting styles: (1) aerial, which means that the individual hunts from the sky; (2) terrestrial, which means that the individual obtains food while walking; (3) insessorial, which means that the individual perches above the ground in trees or other raised substrates; (4) aquatic, which means that the individual obtains its food while being afloat or by diving underwater; and (5) generalist, which means that the individual does not have a particular hunting style but uses different techniques to obtain its food. Falcon species mostly use an aerial, a general or an insessorial hunting style (Tobias et al. 2022). According to the solar glare hypothesis, individuals who experience the sun's glare during hunting (aerial hunters) should have a larger and more intense malar stripe than individuals who experience less sun during hunting (general and insessorial hunters). This was also suggested by Yosef et al. (2012), who demonstrated that the dark plumage of the face of the Masked Shrike ( *Lanius nubicus* ) allows the bird to hunt while facing the sun. Whether hunting style also affects the malar stripe of falcons remains to be tested.

**FIGURE 1:**
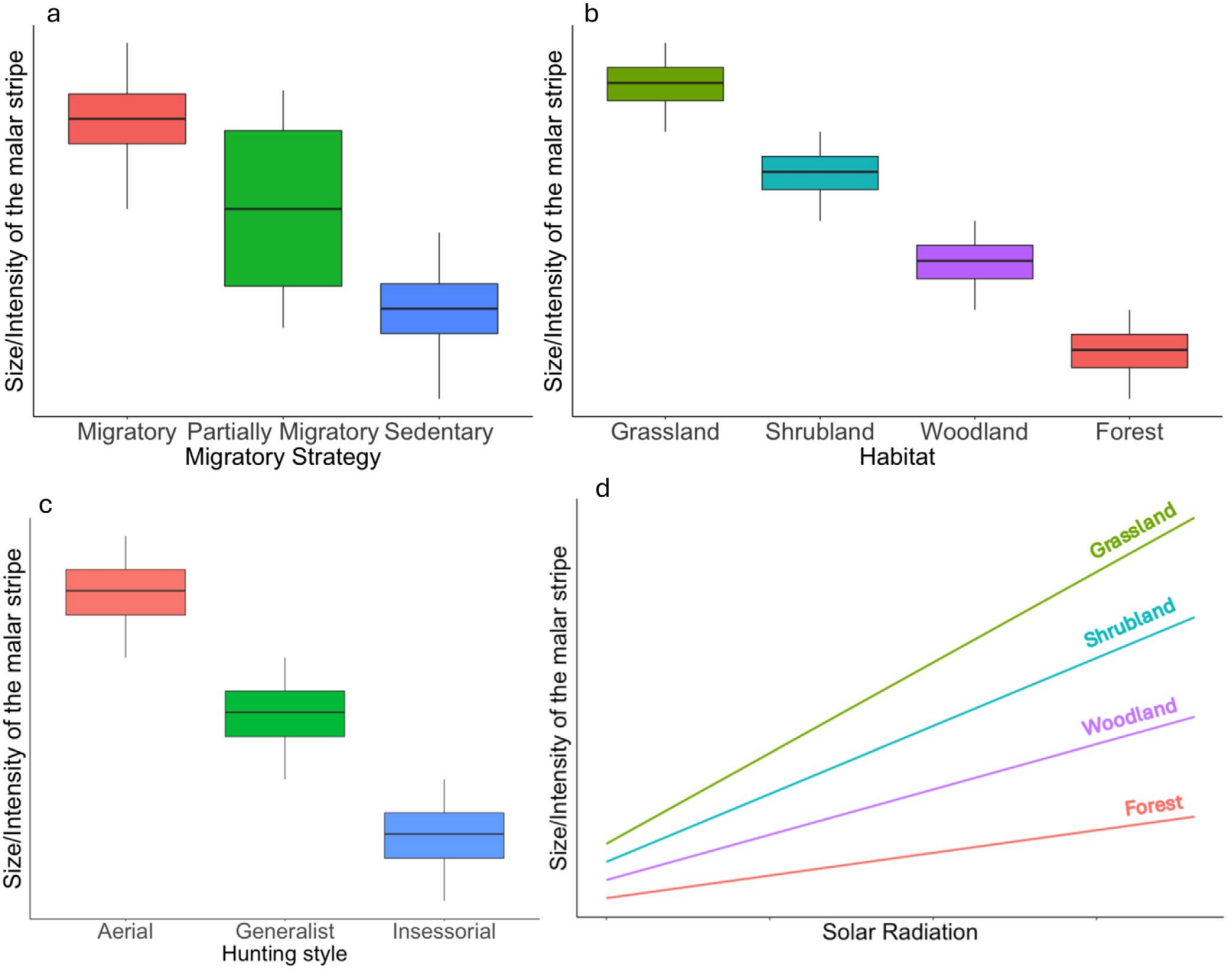
Conceptual graphs with expected patterns. (a) Migratory species are expected to experience more sunlight throughout their annual cycle; hence, their malar stripe is expected to be larger and more intense than the stripes of sedentary species. Partially migratory species consist of both migratory individuals and sedentary individuals; therefore, the malar stripe's sizes and intensities are expected to be in between the migratory and sedentary species. (b) The light intensity of a forest is lower compared to the light intensity of open grasslands. Therefore, individuals living in open grasslands are expected to have a larger and more intense malar stripe than individuals inhabiting forests. (c) The size and intensity of the malar stripe are expected to be larger and more prominent for species with an aerial hunting strategy. Species with an insessorial hunting strategy are expected to have the smallest and least prominent malar stripes. (d) The size and intensity of the malar stripe are expected to increase with increasing solar radiation, depending on the habitat the individuals occupy.

In this study, we aim to understand how differences in migratory strategy, habitat, and hunting style impact the malar stripes of falcon species. Following the solar glare hypothesis, we expect migratory species to have darker and larger malar stripes than sedentary species (Figure 1a). Partially migratory species consist of individuals that are both migratory and sedentary. Therefore, we expect these individuals' malar stripes to be smaller than those of migratory individuals and larger than those of sedentary individuals (Figure 1a). Concerning habitat (grassland, shrubland, woodland, and forest), we expect species that live in open areas and thus experience more sunlight to have larger and more intense malar stripes compared to species that hunt in areas with dense vegetation (Figure 1b). We also expect species that hunt in the air to have a larger and more prominent malar stripe than species with other hunting styles (generalist or insessorial) due to them benefiting from less solar glare during hunting (Figure 1c). Finally, we expect positive relationships between the average solar radiation of the region a sedentary individual is photographed in and the size and intensity of their malar stripes, depending on the habitat they occupy (Figure 1d).

## 2 Methods

### 2.1 Study Species

The *Falco* genus contains 39 different species that are distributed all over the world with different migratory strategies, habitats and hunting styles (Tobias et al. 2022; Winkler et al. 2020). For this study, we compared the malar stripes of 12 falcon species with differing migratory strategies (migratory, partially migratory and sedentary), habitats (grassland, woodland, shrubland and forest), and hunting styles (aerial, insessorial and generalist) (Table 1). We chose the species based on them having a visible malar stripe and on the availability of sufficient photos in the online citizen science repositories. We used two online citizen science repositories, iNaturalist (2024) and the Macaulay Library (2024), to gain access to photos of the 12 different falcon species. These citizen science repositories rely on sophisticated tools to identify species, and experienced reviewers evaluate the observations. Nonetheless, there is the possibility that certain pictures were wrongly identified. However, given the large sample size per species, potential misidentifications will not markedly influence our results.

**TABLE 1:** The 12 falcon species included in this study, with their migratory strategy, habitat, and hunting style (Tobias et al. 2022; Winkler et al. 2020).

Scientific name	English name	Migratory strategy	Habitat^a^	Hunting style
*Falco amurensis*	Amur Falcon	Migratory	Grassland	Insessorial
*Falco columbarius*	Merlin	Migratory	Woodland	Aerial
*Falco naumanni*	Lesser Kestrel	Migratory	Grassland	Generalist
*Falco subbuteo*	Eurasian Hobby	Migratory	Woodland	Aerial
*Falco cenchroides*	Australian Kestrel	Partially Migratory	Woodland	Generalist
*Falco biarmicus*	Lanner Falcon	Partially Migratory	Shrubland	Aerial
*Falco sparverius*	American Kestrel	Partially Migratory	Grassland	Insessorial
*Falco tinnunculus*	Eurasian Kestrel	Partially Migratory	Shrubland	Aerial
*Falco berigora*	Brown Falcon	Sedentary	Woodland	Aerial
*Falco chicquera*	Red‐necked Falcon	Sedentary	Woodland	Aerial
*Falco novaeseelandiae*	New Zealand Falcon	Sedentary	Forest	Aerial
*Falco rufigularis*	Bat Falcon	Sedentary	Forest	Aerial

^a^ Different habitat types (Tobias et al. 2022): Grassland: open grass‐dominated landscape. Shrubland: low stature bushy landscape. Woodland: medium stature tree‐dominated landscape. Forest: tall tree‐dominated landscape.

### 2.2 Analysis of the Photos

The analysis of the photos was based on the methods of Vrettos et al. (2021) and Vrettos (2023). For each species, we analysed 500 photos that were equally distributed over its distribution range. We only included photos that portray adults, and since it is often difficult to determine the sex of an individual, we did not include this factor in the analyses. We randomly selected the photos per species by numbering each photo and using a random generator to determine which photos would be included in the analysis. If we could not analyse a photo, we replaced that photo with a new randomly picked photo. We repeated this procedure until we had 500 good‐quality photos per species. For each photo, we noted down the country, region, GPS coordinates and date it was taken. To avoid including one individual twice, we did not include photos taken in the same location within 5 years of each other. This timeframe is based on the territorial fidelity of falcons (Mcdonald et al. 2003; Zuberogoitia, Martínez, et al. 2009). If there were multiple individuals portrayed in one photo, we analysed all individuals and included them in the analysis.

**FIGURE 2:**
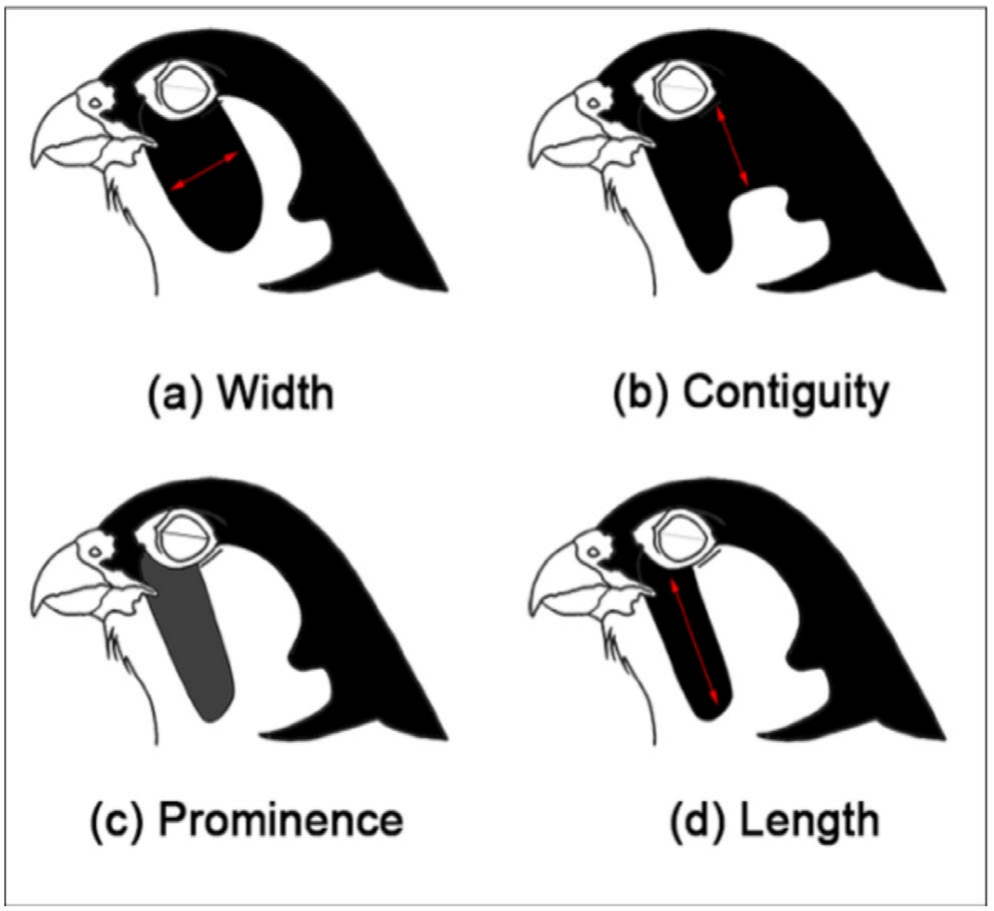
The four aspects of the malar stripe that are measured per individual: The width (a), contiguity (b), prominence (c), and length (d) (Vrettos et al. 2021).

Per individual, we measured and calculated six measurements of the malar stripe: the width of the malar stripe (Figure 2a), which is the thickness of the stripe; the contiguity with the hood (Figure 2b), which is the connection of the malar stripe to the dark plumage on the hood; the prominence (Figure 2c), which is the intensity of the malar stripe; the length of the malar stripe (Figure 2d) which is the maximum distance from the dorsal to the ventral end of the stripe; the elongation, which is calculated by dividing the length by the width; and the surface, which is calculated by multiplying the width by the length.

The width and length were measured using an on‐screen measuring tool (Aequo) (Seager 2022), and these two measures were used to calculate the elongation and surface of the malar stripe. The contiguity and prominence were scored using a scoring template (Figure S1). We also scored the width and length of the malar stripe and used these scores to calculate a scored elongation and surface (Figure S1). Because the position of the birds varied a lot throughout the photos, the measurements were done relative to the width and height of the bird's eye. We performed each measurement three times and used the average of these measurements in the analysis. Also, the bird's posture was scored using a scoring template (Figure S2).

To ensure we were not biased when we scored the photos, we cropped the photos to include only the bird's head, and we scored the photos randomly without knowing their geographical location or the species pictured in them. We also divided the photos of the different species by making groups of 120 photos (10 photos per species) to minimize the influence of analyzing the same species for an extended period. We mixed the photos randomly in these groups of 120 photos to analyze the different species in a mixed order.

### 2.3 Direct Normal Irradiation

We obtained the average direct normal irradiation (kWh/m^2^) using a Global Solar Atlas (Solargis 2024) for each region a photo was taken in. The Handbook of Energy, Section 10–Solar (2013) stated that “Direct Normal Irradiation (DNI) is the amount of solar radiation received per unit area by a surface that is always held perpendicular (or normal) to the rays that come in a straight line from the direction of the sun at its current position in the sky.” Hence, we used direct normal irradiation as a proxy for the amount of sunlight an individual experiences throughout the year.

### 2.4 Statistical Analysis

To test whether there are differences in malar stripes between the falcon species with contrasting migratory strategies, habitats, and/or hunting styles, we first performed a principal component analysis (PCA) to reduce the number of response variables using the built‐in R function *prcomp* (R Core Team 2013). Following the reduction of response variables, we analysed the PC1 and PC2 coordinates using a linear mixed model. For this analysis, we used the lme4 package (Bates et al. 2015) and the car package (Fox and Weisberg 2019) in R (R Core Team 2013) to obtain the *p*‐values. In these models, we included migratory strategy, habitat, and hunting style as fixed factors and the posture variables (horizontal head rotation, vertical head rotation, anterior–posterior head rotation, and head compression), species, and date the photo was taken as random factors. The calculation of variance inflation factor (VIF) values indicated high multicollinearity when the three fixed factors, migratory strategy (VIF = 5.82), habitat (VIF = 22.72), and hunting style (VIF = 7.64), were all included in the model. Therefore, we created two models: Model 1 included migratory strategy (VIF = 3.33) and habitat (VIF = 3.33) and Model 2 included migratory strategy (VIF = 1.12) and hunting style (VIF = 1.12). In each model, we included an interaction effect between the two fixed variables. However, none of the interaction effects were significant, so we removed them from the models.

We used a similar approach (PCA in combination with linear mixed models) to analyze the relationship between the average direct normal irradiation and the different traits of the malar stripes. We used the photo's location to determine the average direct normal irradiation an individual experiences throughout the year. However, we did not know the year‐round locations of the (partially) migratory species because they migrate between wintering and breeding grounds. Therefore, we only included the sedentary species in this analysis: the Bat Falcon ( *Falco rufigularis* ), the Brown Falcon ( *Falco berigora* ), the New Zealand Falcon ( *Falco novaeseelandiae* ), and the Red‐necked Falcon ( *Falco chicquera* ). Next to the overall analysis of the relationships between malar strip characteristics and solar radiation for the sedentary species, we also analyzed the relationship between direct normal irradiation and the different traits of the malar stripe separately for each sedentary species included in this study.

To create the graphs, we used the ggplot2 package in R (Wickham 2016).

## 3 Results

### 3.1 Photos

After processing 6000 different photos, 500 for each species, the total dataset included 6134 individuals across the 12 species due to some photos showing more than one individual. The number of individuals per species ranged from 500 to 526 (Table S1). The individuals included in this study were distributed over 151 countries worldwide (Figure S3). The oldest picture was taken in July 1969, and the most recent in October 2023. For each individual, we measured different aspects of the malar stripe. We used this information to determine if there was a relationship between the size and intensity of the malar stripe and the migratory strategy, the species' habitat or the species' hunting style. The malar stripe characteristics did not change over the period in which the photos were taken (Table S2).

### 3.2 Migratory Strategy, Habitat, and Hunting Style

**FIGURE 3:**
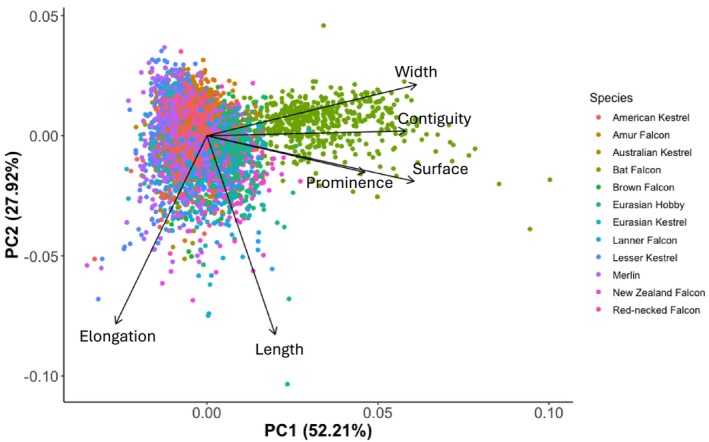
Principal Component Analysis plot based on the different species included in this study. The first PC axis (52.21%) aligns with the width, continuity, surface, and prominence of the malar stripe, whereas the second PC axis (27.92%) algins with the elongation and length of the malar stripe.

The principal component analysis (PCA) showed that the PC1 axis (52.21%) correlated with the width, contiguity, surface, and prominence of the malar stripes, and the PC2 axis (27.92%) correlated with the elongation and length of the malar stripes (Figure 3). We found no significant effect of migratory strategy, habitat, or hunting style on the malar stripes of the falcon species (Table 2). These results were corroborated by additional analysis of the separate measurements and the scored characteristics (Figure S4 and Table S3).

**TABLE 2:** The relationships between the reduced measurements (PC1 and PC2) of the malar stripe and migratory strategy, habitat, or hunting style, including the model per variable, test statistic, and *p*‐value.

Model	Variable	PC1		PC2	
		*X* ^2^	*p*	*X* ^2^	*p*
Model 1	Migratory strategy	0.167	0.920	5.063	0.167
	Habitat	0.396	0.821	2.361	0.501
Model 2	Migratory strategy	2.186	0.335	1.111	0.574
	Hunting style	0.589	0.753	1.672	0.433

*Note:* All variables could not be analyzed in one model due to high collinearity between habitat and hunting style.

### 3.3 Direct Normal Irradiation

To test for a relationship between the average direct normal irradiation and the size and intensity of the malar stripe, we created a subset of the dataset including only sedentary species, namely the Bat Falcon, the Brown Falcon, the New Zealand Falcon, and the Red‐necked Falcon. Sedentary species stay in the same location year‐round. Therefore, the average direct normal irradiation of the location a sedentary individual is photographed in is representative of the average direct normal irradiation that the individual experiences throughout the year. The sedentary species in this analysis occupied forest (Bat Falcon and New Zealand Falcon) and woodland habitats (Brown Falcon and Red‐necked Falcon).

**FIGURE 4:**
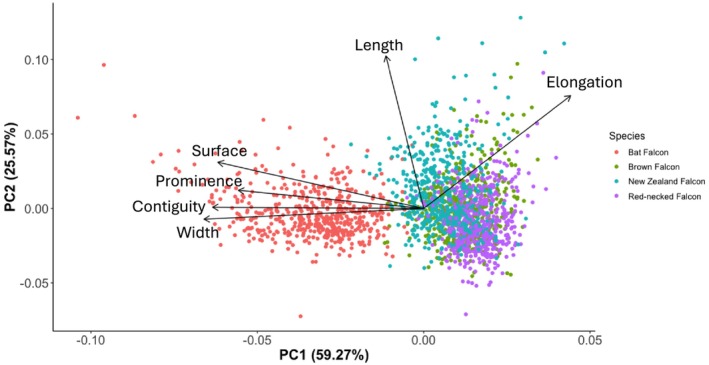
Principal Component Analysis of the sedentary species included in this study. The first PC axis (59.27%) aligns with the width, continuity, surface, and prominence of the malar stripe, whereas the second PC axis (25.57%) aligns with the elongation and length of the malar stripe.

After performing a Principal Component Analysis, we found that the PC1 axis (60%) correlated with the surface, prominence, contiguity, and width of the malar stripe (Figure 4). The PC2 axis (25%) correlated with the length and elongation of the malar stripe (Figure 4). The relationships between the PC1 axis and solar radiation depended on the habitat the individual occupied (*X* ^2^ = 23.222, *p*‐value < 0.001; Table 3) and on the species (*X* ^2^ = 11.871, *p*‐value < 0.001; Table 3). These results were also corroborated by additional analysis of the separate measurements and the scored characteristics (Figure S5 and Table S4).

**TABLE 3:** The relationships between the principal component axis and the average direct normal irradiation (solar) of the location where the photo is taken, including interaction effects between habitat and direct normal irradiation and species and direct normal irradiation.

Variable	Model	Habitat		Solar		Species		Habitat × Solar		Solar × Species	
		*X* ^2^	*p*	*X* ^2^	*p*	*X* ^2^	*p*	*X* ^2^	*p*	*X* ^2^	*p*
PC1	LMM	2146.554	**< 0.001**	3.274	0.070	3588.50	**< 0.001**	23.222	**< 0.001**	11.871	**< 0.001**
PC2	LMM	2486.321	**< 0.001**	3.226	0.073	3572.764	**< 0.001**	—	—	—	—

*Note:* The table includes per measurement: the type of model, the test statistic, and *p*‐value for each fixed factor and the interaction effects. Significant results (*p*‐value < 0.05) are highlighted in bold.

**FIGURE 5:**
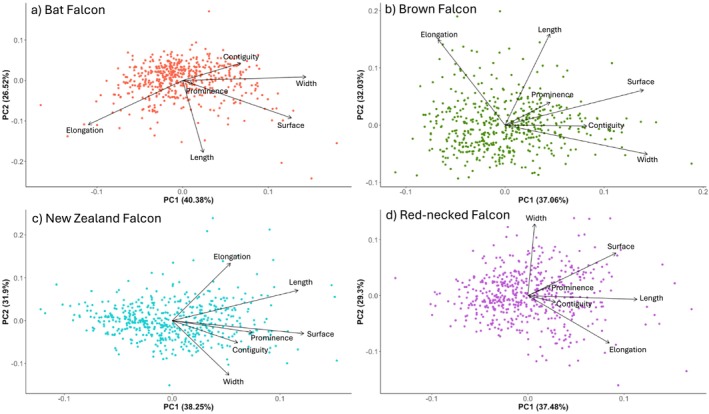
Principal Component Analysis of the four sedentary species separately, (a) Bat Falcon, (b) Brown Falcon, (c) New Zealand Falcon, (d) Red‐necked Falcon. See main text for details on each species.

Because the relationships between the measurements of the malar stripe and the average direct normal irradiation differed per species, we also analyzed the relationship between the measurements of the malar stripe and solar radiation for each species separately using a principal component analysis (Figure 5) in combination with a linear mixed model (Table 4). All scored measurements of the malar stripe were also analyzed separately (Figures S6–S8 and Table S5).

**TABLE 4:** The relationships between the principal component axis and the average direct normal irradiation per species, including the model type, the estimates per model, the test statistic, and the *p*‐value.

Variable	Bat Falcon				Brown Falcon				New Zealand Falcon				Red‐necked Falcon			
	Model	Estimate	*t*	*p*	Model	Estimate	*t*	*p*	Model	Estimate	*t*	*p*	*Model*	*Estimate*	*t*	*p*
PC1	LMM	−0.297	−3.808	**< 0.001**	LMM	0.293	4.170	**< 0.001**	LMM	−0.107	−1.287	0.198	LMM	0.153	2.456	**0.014**
PC2	LMM	0.024	0.404	0.686	LMM	−0.041	−0.700	0.484	LMM	−0.112	−1.516	0.130	LMM	0.113	2.074	**0.038**

*Note:* Significant results (*p*‐value < 0.05) are highlighted in bold.

For the Bat Falcon, the PC1 axis (40.38%), which correlated with the contiguity, width, prominence, and surface (Figure 5a), had a significant relationship with the amount of solar radiation in the area the photo was taken (LMM, *t* = −3.808, *p*‐value < 0.001, Table 4). The relationship between the PC2 axis (26.52%), which correlated with the elongation and the length, and solar radiation was not significant (LMM, *t* = 0.404, *p*‐value < 0.686, Table 4).

The Brown Falcon showed a significant relationship between the PC1 axis (37.06%) (Figure 5b) and the solar radiation (LMM, *t* = 4.170, *p*‐value < 0.001, Table 4). The PC1 axis correlated with the contiguity, width, prominence, and surface of the malar stripe. The relationship between the PC2 axis (32.03%), which correlated with the elongation and length of the malar stripe, and the solar radiation, on the other hand, was not significant (LMM, *t* = −0.700, *p*‐value = 0.484, Table 4).

The measurements of the malar stripes of the New Zealand Falcon correlated with the PC1 axis that explained 38.25% of the variation (Figure 5c). There was no significant relationship between this axis and the solar radiation (LMM, *t* = −1.287, *p*‐value 0.198, Table 4). Also, the relationship between the PC2 axis (31.9%) and solar radiation was not significant (LMM, *t* = −1.516, *p*‐value = 0.130, Table 4).

All measurements, besides the width, of the malar stripes of the Red‐necked falcon correlated with the PC1 axis (37.48%) which had a significant relationship with the amount of solar radiation of the location where the photo was taken (LMM, *t* = 2.456, *p*‐value = 0.014, Table 4 and Figure 5d). The PC2 axis (29.3%) correlated with the width of the malar stripe and also had a significant relationship with solar radiation (LMM, *t* = 2.074, *p*‐value = 0.038, Table 4).

In summary, each species showed a different relationship between the size and intensity of its malar stripe and the amount of solar radiation. Hence, no clear pattern could be observed.

## 4 Discussion

According to the solar glare hypothesis, the malar stripe should be more prominent and larger with increasing sunlight. We analyzed the malar stripe of 12 falcon species by scoring and measuring different malar stripe characteristics. Our results show that the malar stripes of species did not differ significantly between species with differing migratory strategies, habitats, and hunting styles. However, for several sedentary species (Bat Falcon, the Brown Falcon and the Red‐necked Falcon), most malar stripe measurements were significantly related to the amount of solar radiation, providing some support for the solar glare hypothesis.

### 4.1 Migratory Strategy

The malar stripes of individuals with different migratory strategies did not differ. This pattern differs from what we hypothesized since we expected migrating individuals to experience more sunlight throughout the year and, therefore, have a larger and more prominent malar stripe. Vrettos et al. (2021), who analyzed the malar stripes of the partially migratory Peregrine Falcon, included a repeat analysis in their study in which they only included non‐migratory subspecies and photos taken in the breeding season. They found no changes to their statistical relationships, suggesting no differences between migrating and non‐migrating individuals.

A possible reason for the lack of differences between malar stripes of species with varying migratory strategies could be that the assumption that the species included in this study experienced different amounts of sunlight throughout the year depending on their migratory strategy was not valid. Migrating individuals experience approximately constant daylight hours throughout the year because they move to areas with more daylight hours when the days become shorter in their current location (Berthold 1996; Kok et al. 1990; Sockman and Hurlbert 2020). The sedentary species included in this study were primarily located around the equator, where the amount of daylight hours is also approximately constant throughout the year (Forsythe et al. 1995), or in Australia, which has a high number of sunlight hours year‐round (Harlfinger 1993). Hence, all species in this study probably experienced approximately constant daylight hours throughout the year.

To compare individuals who experience different numbers of daylight hours in future studies, we suggest including species that occur further away from the equator, such as the Madagascar Kestrel ( *Falco newtoni* ) (Kemp and Kirwan 2021), the Greater Kestrel ( *Falco rupicoloides* ) (Kemp and Kirwan 2020b) or the Dickinson's Kestrel ( *Falco dickinsoni* ) (Kemp and Kirwan 2020a), so that individuals who experience a different average number of daylight hours can be compared. Also, in this study, the partially migratory individuals were not separated between sedentary and migratory individuals. Partially migratory individuals found at their breeding ground during the winter could be considered permanent residents and, thus, sedentary (Henny and Brady 1994; Holte et al. 2016). Individuals found at a location different from their breeding ground during the winter could be considered migratory (Henny and Brady 1994; Holte et al. 2016). By separating sedentary and migratory individuals, one could compare the malar stripes of individuals of the same species with differing migratory strategies.

### 4.2 Habitat

The malar stripes of individuals inhabiting different habitats did not differ significantly for most measurements. This is not in line with the solar glare hypothesis. We expected that individuals living in areas with a high tree cover percentage, such as forest or woodland, would have smaller and less intense malar stripes than those living in areas with a lower tree cover percentage. Vrettos (2023) also did not find positive relationships after accounting for habitat, a pattern that aligns with our results. She did not directly compare malar stripe characteristics of falcon species occupying different habitats, but she did account for habitat when analyzing the relationships between various malar stripe characteristics and solar radiation.

However, our classification into the four habitat types, forest, woodland, shrubland, and grassland, might have been too simplistic. The falcon species in this study live in semi‐open to open habitats (Tobias et al. 2022). For forest species, this means that they reside primarily above the canopy. In addition, the two forest species included in this study, the Bat Falcon and the New Zealand Falcon, are mostly found on the edges of forests and in fragmented forests, such as riverbanks or agricultural fields with some trees (del Hoyo et al. 1994; Ferguson‐Lees and Christie 2001; Horikoshi et al. 2017; Seaton et al. 2013). Also, we only included one habitat type per species, whereas falcon species could inhabit more than one type of habitat (Orta et al. 2020a). Hence, even though species live in different habitats with different tree cover percentages, they could still experience the same amount of sunlight.

### 4.3 Hunting Style

The hunting style of the individual could also affect the amount of sunlight an individual experiences throughout the day (Elkins 2010). We expected species with an aerial hunting strategy to have larger and more intense malar stripes compared to generalists, and we expected that the malar stripes of species with an insessorial hunting strategy would have the smallest and palest malar stripes. However, we found no differences in malar stripe characteristics between species with differing hunting styles.

Dark markings around the eyes have been observed in different avian and non‐avian species with varying hunting strategies (Caro 2009; Ortolani 1999). Vrettos (2023) suggested that the malar stripes of falcons do not enhance the sighting of prey since she found no relationship between prey preference and malar stripe characteristics, which is in line with our results. On the other hand, Yosef et al. (2012) found increased hunting success for Masked Shrikes ( *Lanius nubicus* ), which hunt facing the sun, with darker face masks. The Ring‐tailed Lemur ( *Lemur catta* ) also has dark eye markings, which are suggested to have an anti‐glare function (Caro 2009), while they browse trees to find leaves and fruit (Mertl‐Millhollen et al. 2003). Finally, Ortolani (1999) suggested that the dark eye markings in carnivores are possibly used to reduce the sun's glare in the eyes of the animal. These examples suggest that other animal species do possibly use their facial markings to reduce the sun's glare in their eyes during hunting.

However, not all falcon species hunt in direct sunlight. For example, the Amur Falcon, the Bat Falcon, the Eurasian Hobby, and the Lanner Falcon have also been observed hunting during dusk or dawn (del Hoyo et al. 1994; Ferguson‐Lees and Christie 2001; Kemp and Marks 2020; Orta et al. 2020b; Stanton 2016; Bryson, 2022). The Australian Kestrel, the Lesser Kestrel, and the Eurasian Hobby are all observed hunting by moonlight (Couzens 2015; del Hoyo et al. 1994; Ehrlich et al. 1988). These species all have a malar stripe, which suggests that malar stripes might have a different function than the one suggested by the solar glare hypothesis.

### 4.4 Direct Normal Irradiation

Sedentary species stay in one place year‐round. Hence, the average direct normal irradiation of their location represents the amount of sunlight individuals experience throughout the year. Therefore, we only analyzed the relationships between malar stripe characteristics and direct normal irradiation for the sedentary species in this study. Most of the relationships between the malar stripe characteristics and direct normal irradiation significantly depended on the habitat the individual occupied and the species.

All the significant relationships that depended on the individual's habitat were positive, meaning that the size and intensity of the malar stripe increased with increasing solar radiation, except for the malar stripe length of the woodland species. This pattern aligns with the solar glare hypothesis that predicts larger malar stripes in areas with higher solar radiation (Vrettos et al. 2021).

There was interspecific variation in the relationships between certain malar stripe measurements and solar radiation. However, no clear pattern could be observed between the different species. Vrettos (2023) also found different relationships between the size and intensity of the malar stripe and solar radiation for the different species included in her study. The Eleonora's Falcon ( *Falco eleonorae* ), the Madagascar Kestrel and the Gyrfalcon ( *Falco rusticolus* ) had smaller malar stripes in regions with higher solar radiation. On the other hand, Dickinson's Kestrels, Rock Kestrels ( *Falco rupicolus* ), and Peregrine Falcons had larger malar stripes in regions with higher solar radiation. The different relationships between malar stripe characteristics and solar radiation between species suggest that there might be other explanations for the size and intensity of the malar stripe than the solar glare hypothesis.

### 4.5 Alternative Explanations

The various characteristics of the malar stripe did not differ between species with different migratory strategies, habitats, and hunting styles. This suggests that the malar stripe might have other functions than reducing the sun's glare, such as thermoregulation (Rogalla et al. 2022; Wolf and Walsberg 2000) or camouflage (Galeotti et al. 2003; Johnson and Burnham 2011; Tate and Amar 2017; Tate et al. 2016).

An explanation for the negative correlation between some measurements of the malar stripe of certain species and the direct normal irradiation could be that the plumage colour of those species functions in the animal's thermoregulation (Rogalla et al. 2022; Wolf and Walsberg 2000). According to the thermal melanism hypothesis, individuals with a darker colouration absorb more solar radiation than those with a lighter colouration (Watt 1968). A study by Fargallo et al. (2018) showed that Griffon Vultures ( *Gyps fulvus* ) that grow up in nests that are exposed to direct sunlight develop a lighter plumage colour compared to individuals that grow up in nests that are less exposed to direct sunlight. Galván et al. (2018) also reported that individuals with darker plumage colouration are restricted to colder environments by comparing the plumage colouration of 96 bird species across their distribution range in Spain, including two falcon species: the Peregrine Falcon and the Eurasian Hobby. So, according to the thermal melanism hypothesis, individuals should have larger and more prominent malar stripes in regions with lower average direct solar irradiation, which is what we observed in Bat Falcons, Brown Falcons, and New Zealand Falcons.

Another explanation for the larger malar stripes in regions with lower average direct normal irradiation could be that those individuals have a higher foraging success due to being less detectable by prey (Galeotti et al. 2003; Johnson and Burnham 2011; Tate and Amar 2017; Tate et al. 2016). This was also suggested by both Vrettos (2023) and Amar et al. (2013), who found lighter morph individuals in areas with high solar radiation and darker‐colored individuals in areas with low solar radiation. It has also been suggested that the malar stripe could function as camouflage for the eye. The eyes of animals surrounded by a dark spot or close to a dark spot might be more difficult to detect (Gavish and Gavish 1981; Josef 2017; Stevens and Merilaita 2009). These alternative explanations remain to be tested with additional analyses. For future research, we recommend expanding the number of falcon species and taking into account phylogenetic relationships to unravel the environmental and evolutionary drivers of the malar stripe in this bird family.

## 5 Conclusion

We found no differences in the measurements of the malar stripe between species with different migratory strategies, hunting styles, and habitats. This lack of differences could be because the falcon species in this study all experience approximately the same amount of sun throughout the year and hence do not develop a malar stripe of a different size or intensity. For sedentary species, the relationships between most of the malar stripe characteristics and solar radiation depended on the species or the habitat they occupied. These patterns suggest that there are other functions of the malar stripe, such as thermoregulation or camouflage. The sex of an individual could potentially also affect the size and intensity of the malar stripe (Hartley et al. 1993; Hudon et al. 2015; Zuberogoitia, Azkona, et al. 2009; Zuberogoitia et al. 2013), but this aspect remains to be investigated in falcons. Therefore, we would recommend that future studies include the individual's sex and test if the relationships between malar stripe characteristics and solar radiation are affected by it.

## Author Contributions

**Celeste Polak:** conceptualization (equal), data curation (lead), formal analysis (lead), methodology (equal), visualization (lead), writing – original draft (lead), writing – review and editing (equal). **Jente Ottenburghs:** conceptualization (equal), formal analysis (supporting), methodology (equal), supervision (lead), visualization (supporting), writing – original draft (supporting), writing – review and editing (equal).

## Conflicts of Interest

The authors declare no conflicts of interest.

## Supporting information


Data S1.

## Data Availability

The data and R‐code that support the findings of this study will be openly available in Dryad at https://doi.org/10.5061/dryad.cc2fqz6gg. The data and R‐code can currently be accessed through a temporary link: http://datadryad.org/stash/share/aL_Z8Kt_gmviqs26‐4SeU8MR_nkmvgu0HY7eArnJBQ4.
