# Supplementary material for: Migration, Habitat and Hunting Style Do Not Affect the Malar Stripe of Different Falcon Species
Source: Ecol Evol. 2025 Apr 27;15(4):e71028. doi: 10.1002/ece3.71028 (PMC12034159; doi:10.1002/ece3.71028)
Supplement: Supplementary file 1 — Data S1. [file ECE3-15-e71028-s001.docx]

**APPENDIX: Supplementary figures and tables**

**Figure S1.** The scoring template used to score (A) malar stripe width, (B) contiguity of the malar stripe with the hood, (C) malar stripe prominence and (D) malar stripe length. From: Vrettos et al. (2021).

**Figure S2.** Scoring scheme to quantify and score head angle and degree of plumage distortion in web-sourced photographs of different falcon species. Head angle variables scored include (a) head orientation in terms of pitch rotation, or rotation around the horizontal axis; (b) head orientation in terms of yaw rotation, or rotation around the vertical axis; and (c) head orientation in terms of roll rotation, or rotation around the anterior-posterior axis. Plumage distortion variables scored include (d) degree of plumage distortion as a result of vertical feather compression due to the bird sitting slumped or hunched, and (e) degree of plumage distortion as a result of ptiloerection. From: Vrettos (2023).


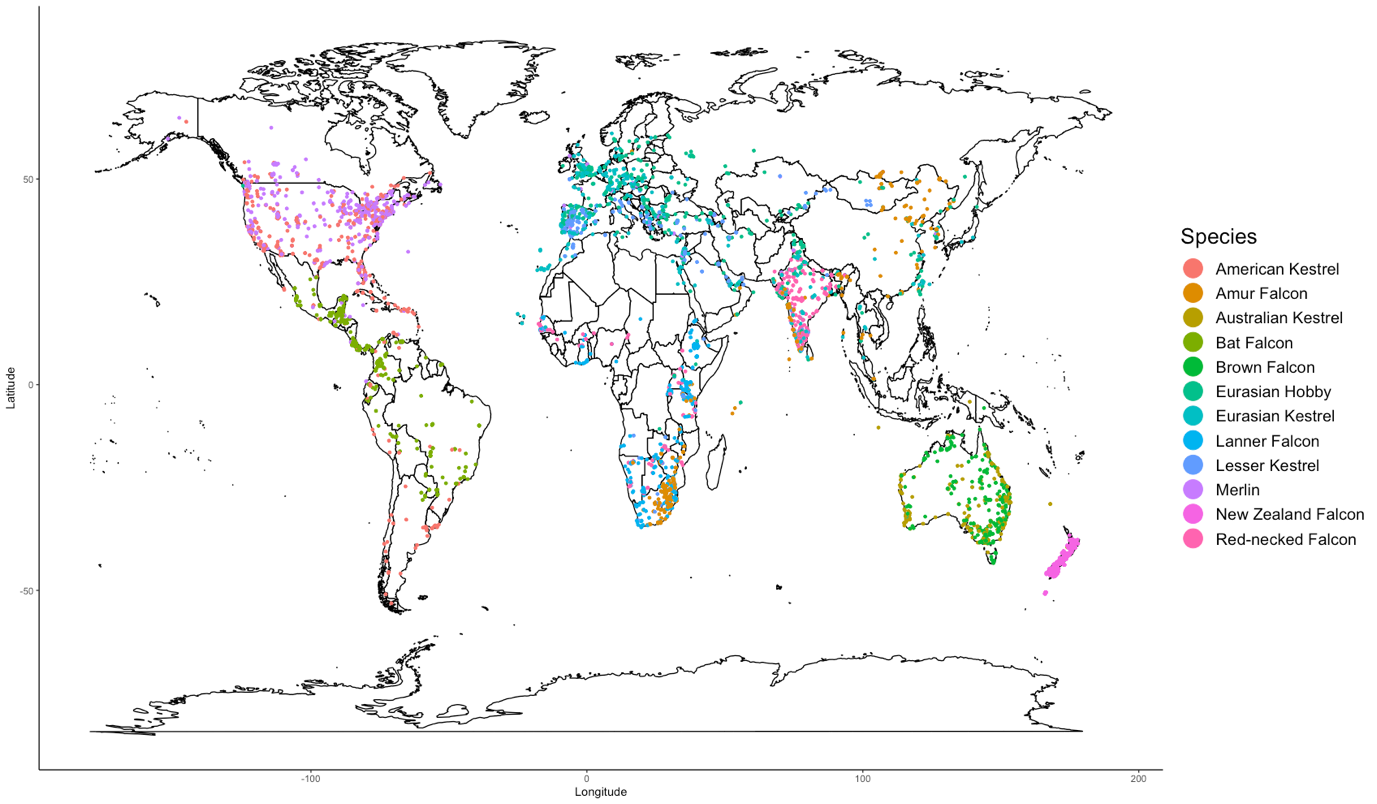


**Figure S3.** Geographical locations of all the open-source pictures used in this study. Different colors correspond to different falcon species.

**Figure S4.** Principal component analysis of the scored measurements of the malar stripe for all falcons species in this study. Different colors correspond to different falcon species.


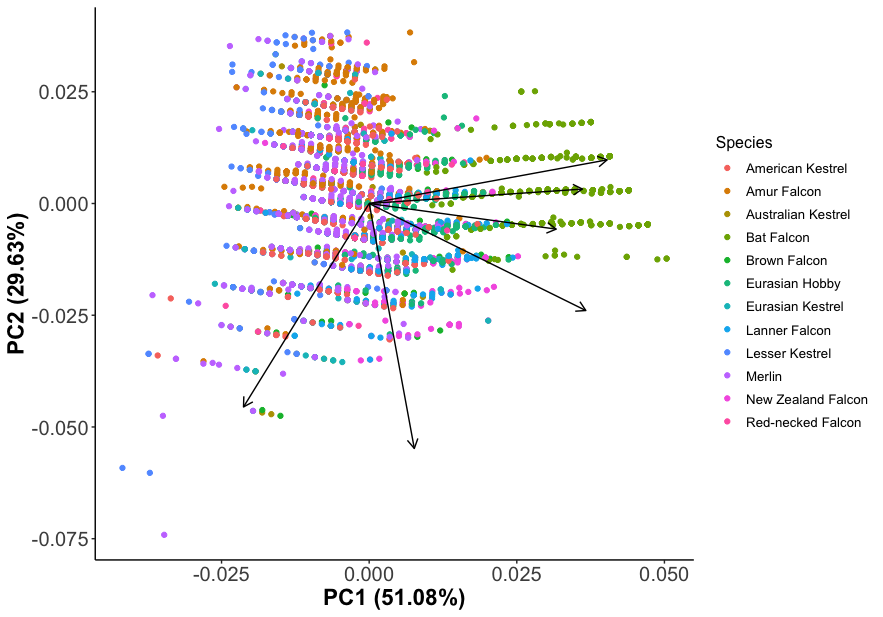


Width

Contiguity

Prominence

Surface

Elongation

Length


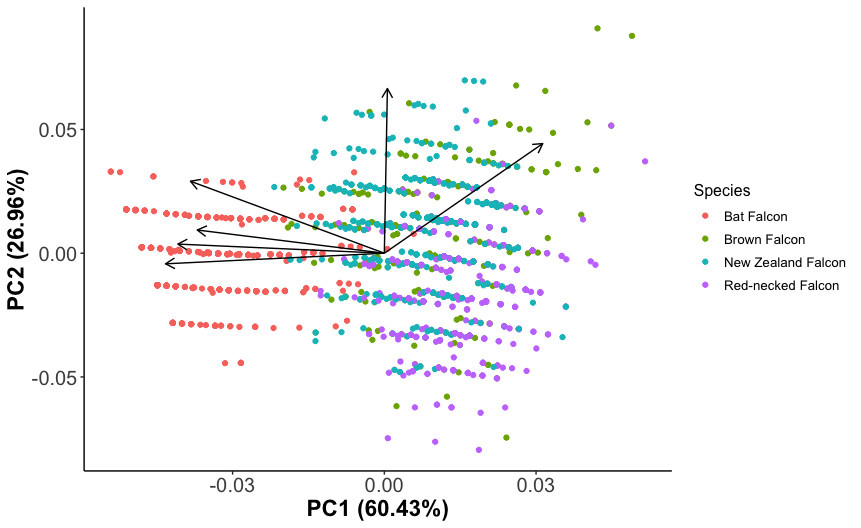


Elongation

Length

Surface

Prominence

Contiguity

Width

**Figure S5.** Principal component analysis of the scored measurements of the malar stripe for the sedentary falcons species in this study. Different colors correspond to different falcon species.


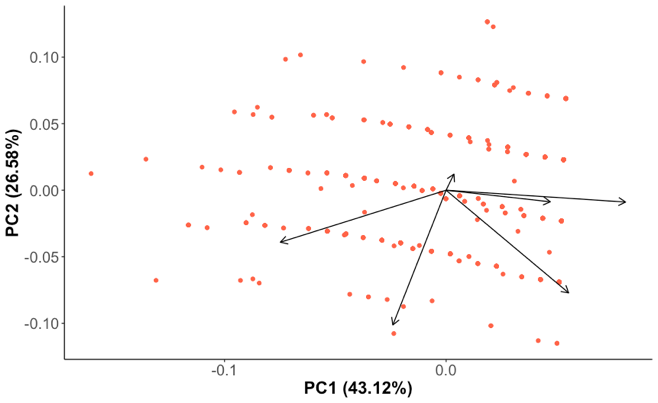


Elongation

Prominence

Surface

Length

Contiguity

Width

A - Bat Falcon


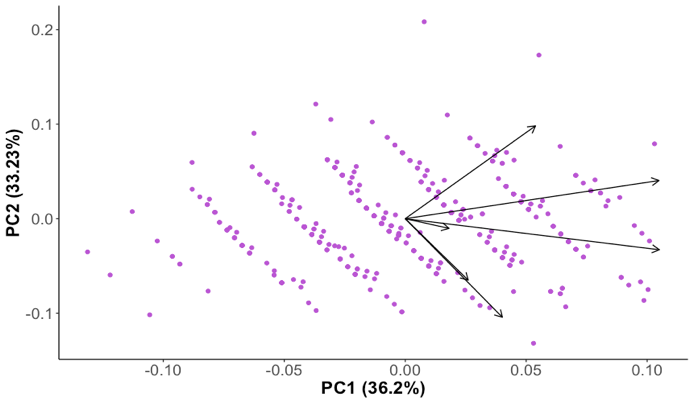


Elongation

Length

Surface

Contiguity

Prominence

Width

D - Red-necked Falcon


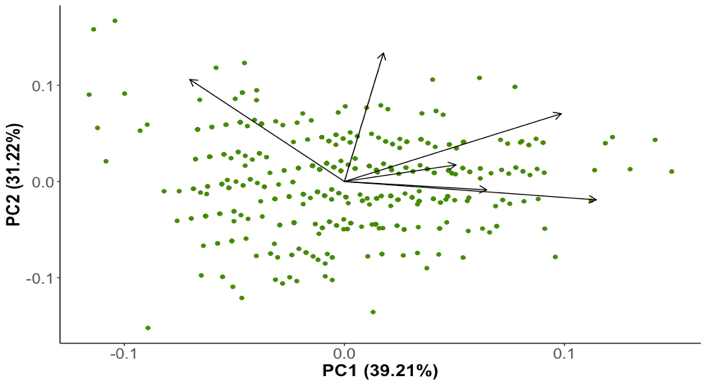


Elongation

Length

Surface

Prominence

Contiguity

Width

B - Brown Falcon


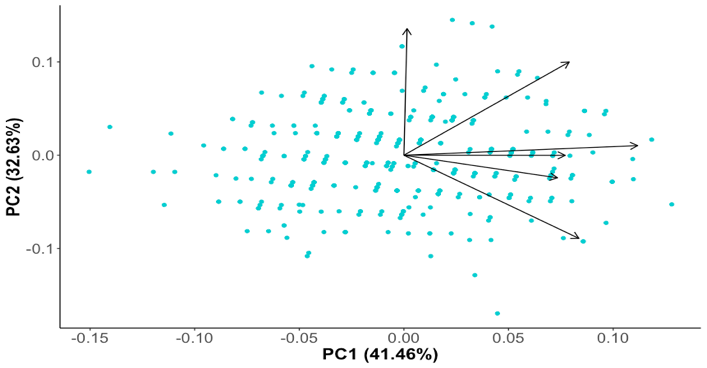


Elongation

Length

Surface

Prominence

Contiguity

Width

C - New Zealand Falcon

**Figure S6.** Separate principal component analyses of the scored measurements of the malar stripe for the four sedentary falcons species in this study.


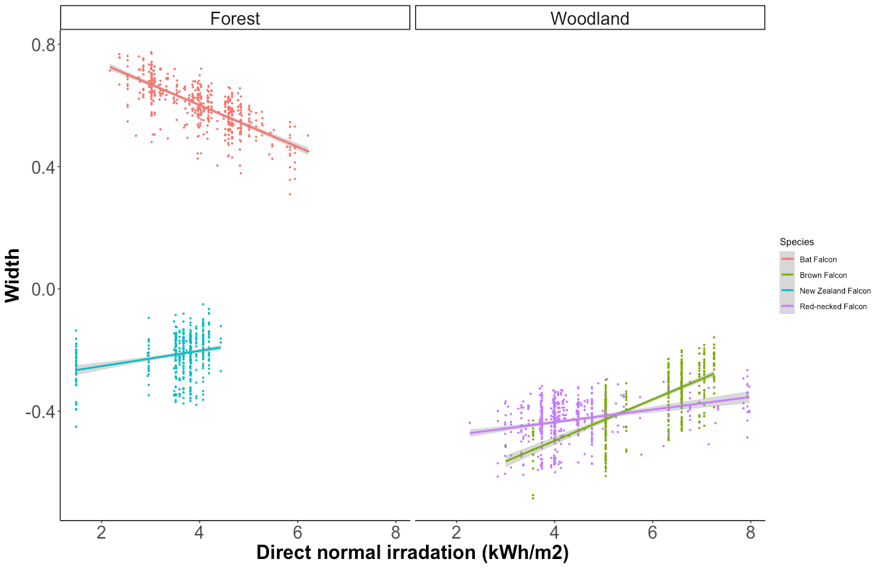

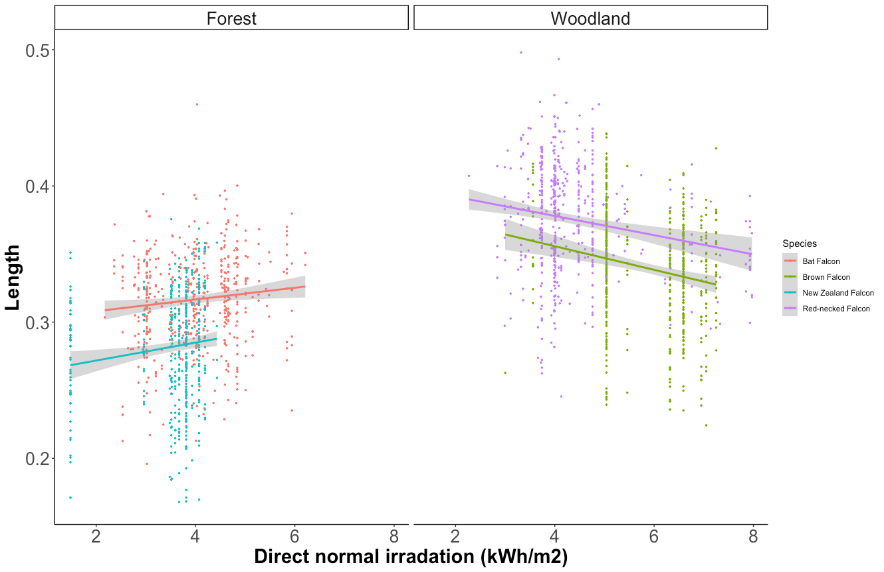

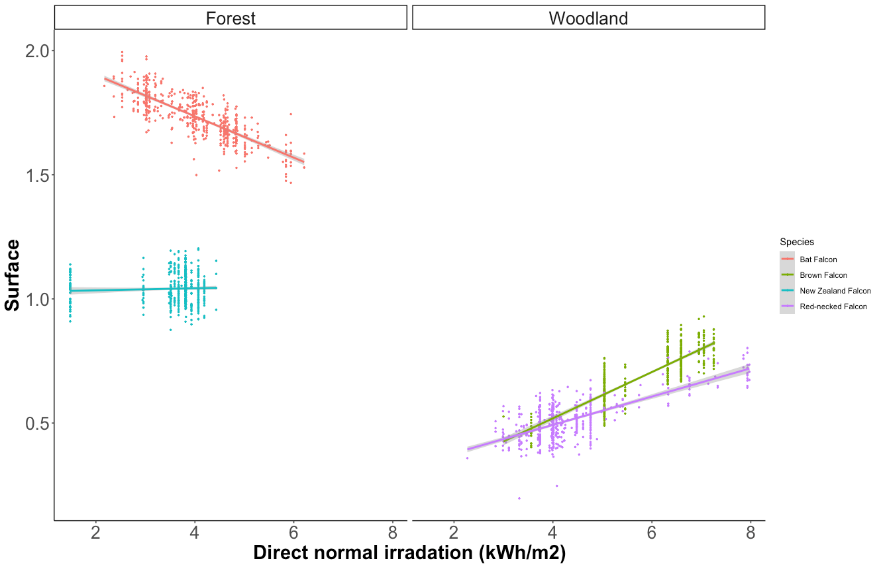

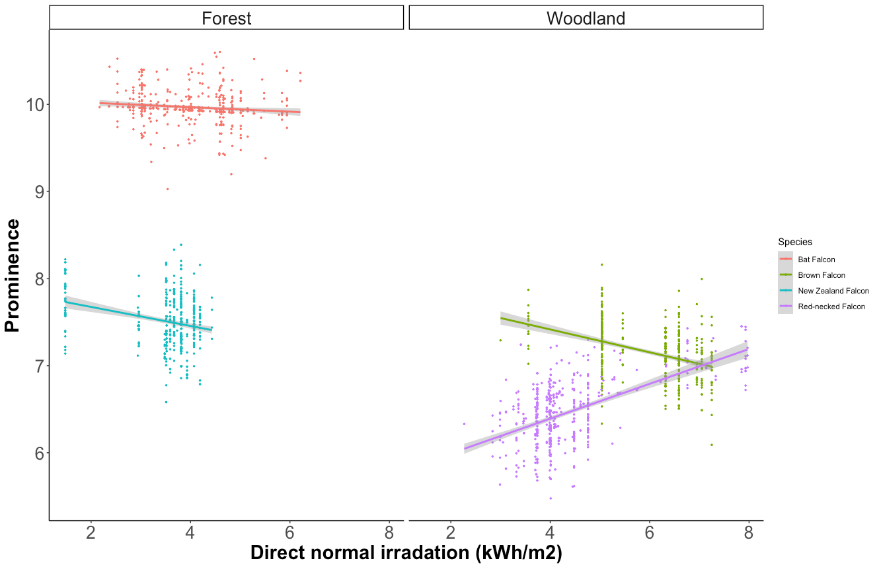

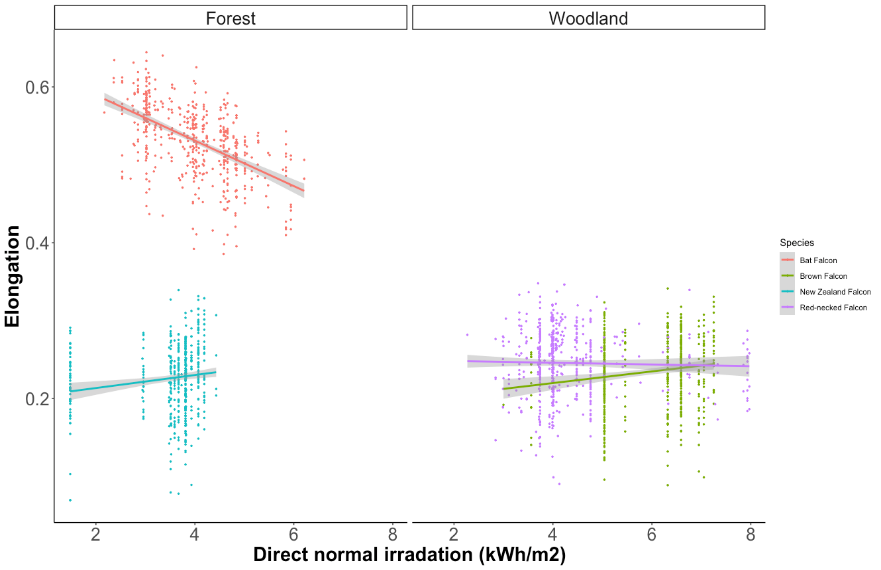

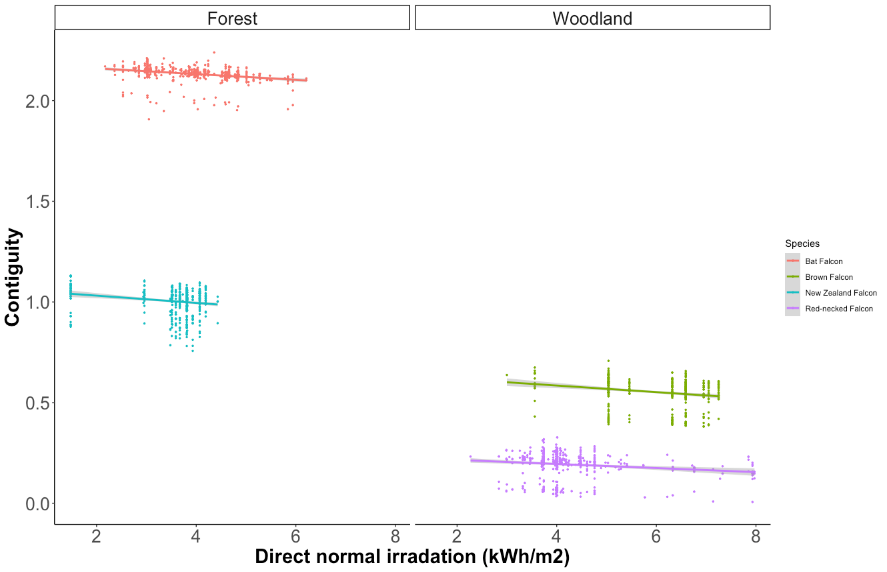


A

B

C

D

E

F


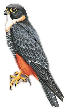

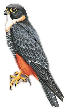

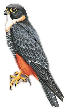

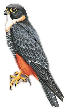

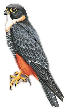

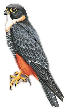

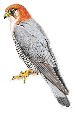

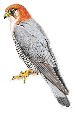

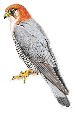

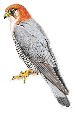

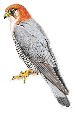

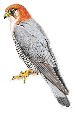

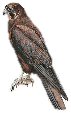

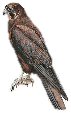

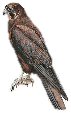

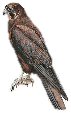

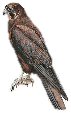

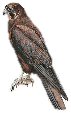

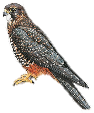

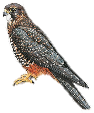

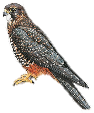

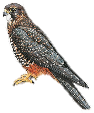

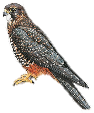

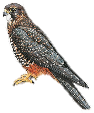


**Figure S7.** Relationships between direct normal irradiation and the different measurements of the malar stripe of the four sedentary species: (a) width, (b) length, (c) elongation, (d) contiguity, (e) prominence, and (f) surface. Drawings used with permission of the Handbook of Birds of the World.


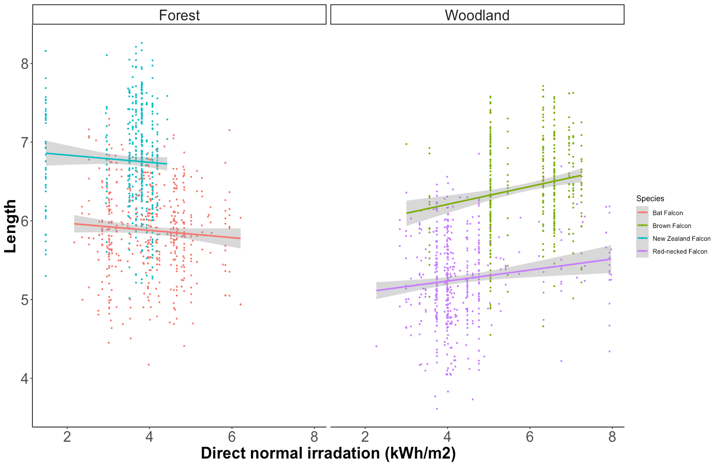

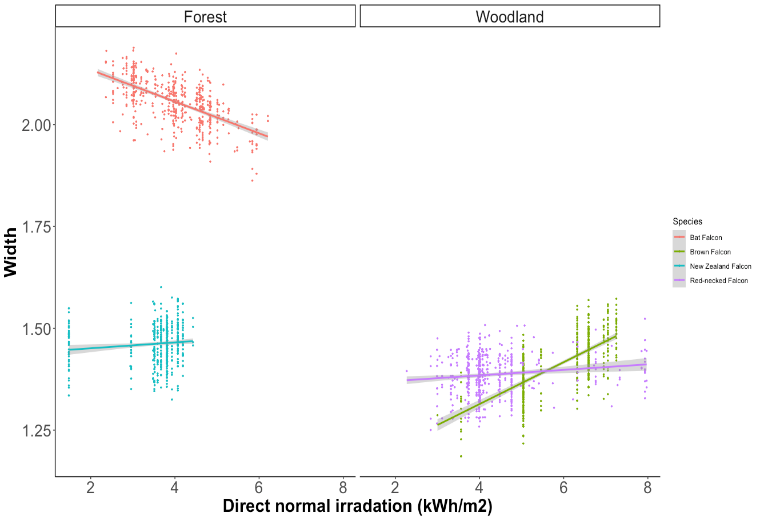


A

B


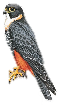

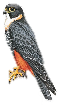

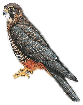

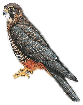

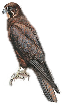

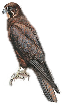

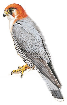

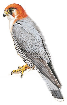


C

D


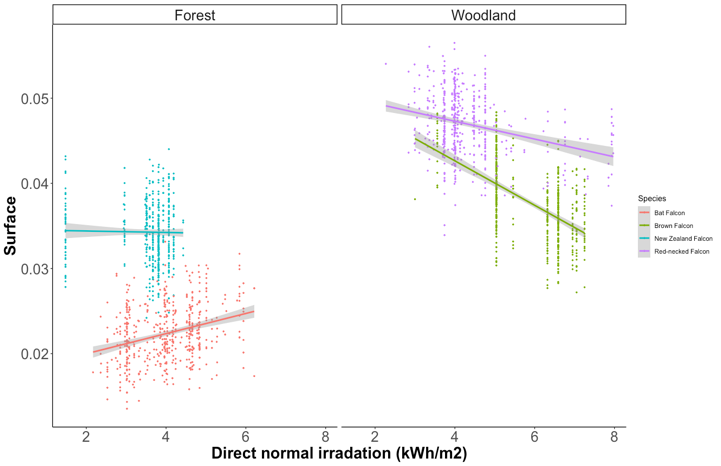

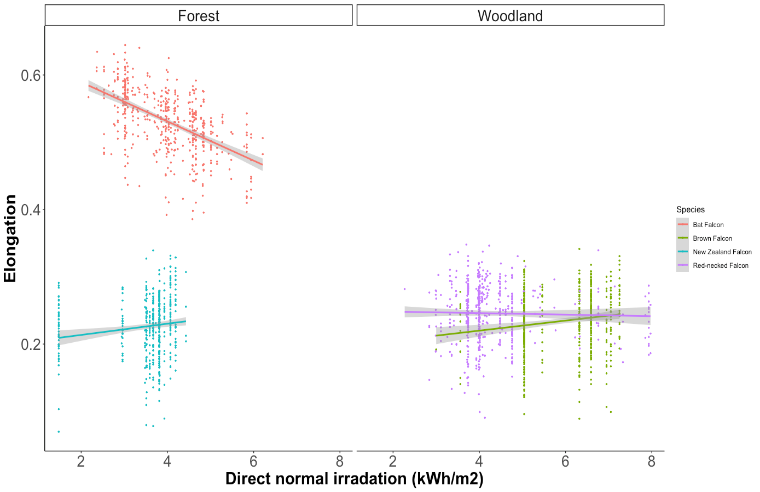

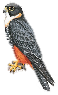

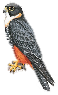

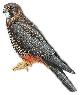

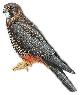

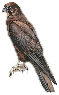

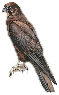

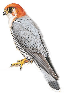

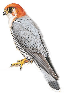


**Figure S8**. Relationships between direct normal irradiation and the different scored measurements of the malar stripe of the four sedentary species: (a) width, (b) length, (c) elongation, and (d) surface. Drawings used with permission of the Handbook of Birds of the World.

**Table S1**. Number of individuals for each species in the study.

| Species | Scientific name | Number of individuals |
| --- | --- | --- |
| American Kestrel | *Falco sparverius* | 511 |
| Amur Falcon | *Falco amurensis* | 521 |
| Australian Kestrel | *Falco cenchroides* | 505 |
| Bat Falcon | *Falco rufigularis* | 519 |
| Brown Falcon | *Falco berigora* | 503 |
| Eurasian Hobby | *Falco subbuteo* | 502 |
| Eurasian Kestrel | *Falco tinnunculus* | 504 |
| Lanner Falcon | *Falco biarmicus* | 516 |
| Lesser Kestrel | *Falco naumanni* | 520 |
| Merlin | *Falco columbarius* | 500 |
| New Zealand Falcon | *Falco novaeseelandiae* | 507 |
| Red-necked Falcon | *Falco chicquera* | 526 |

**Table S2**. Model output for the relationship between the date at which the photo was taken and the malar stripe characteristics (Width, Length, Elongation, Surface, Contiguity, and Prominence).

| **Characteristic** | **Estimate** | **t-value** | **p-value** |
| --- | --- | --- | --- |
| Width | -1.812 * e^-11^ | -0.554 | 0.579 |
| Length | 1.978 * e^-10^ | 1.888 | 0.059 |
| Elongation | 3.003 * e^-10^ | 1.186 | 0.236 |
| Surface | 8.455 * e^-11^ | 0.592 | 0.554 |
| Contiguity | -1.927 * e^-10^ | -1.902 | 0.057 |
| Prominence | -1.324 * e^-10^ | -0.885 | 0.376 |

**Table S3.** Model output for the relationships between the reduced scored measurements of the malar stripe (PC1 and PC2) and three variables (migratory strategy, habitat and hunting style).

|  | | **PC1** | | **PC2** | |
| --- | --- | --- | --- | --- | --- |
| **Model** | **Variable** | **X^2^** | **p-value** | **X^2^** | **p-value** |
| Model 1 | Migratory strategy | 0.016 | 0.992 | 3.882 | 0.144 |
|  | Habitat | 4.210 | 0.240 | 1.807 | 0.613 |
| Model 2 | Migratory strategy | 2.238 | 0.312 | 5.167 | 0.076 |
|  | Hunting style | 1.175 | 0.556 | 0.984 | 0.611 |

**Table S4.** The relationships between the reduced scored measurements of the malar stripe (PC1 and PC2) and migratory strategy, habitat, or hunting style, including the model per variable, test statistic, and p-value.

|  |  | **Habitat** | | **Solar** | | **Species** | | **Habitat * Solar** | | **Solar * Species** | |
| --- | --- | --- | --- | --- | --- | --- | --- | --- | --- | --- | --- |
| **Variable** | **Model** | **X^2^** | **p-value** | **X^2^** | **p-value** | **X^2^** | **p-value** | **X^2^** | **p-value** | **X^2^** | **p-value** |
| PC1 | LMM | 5426.728 | **< 0.001** | 0.066 | 0.797 | 3690.896 | **< 0.001** | 18.339 | **< 0.001** | 9.108 | **0.011** |
| PC2 | LMM | 148.944 | **< 0.001** | 0.081 | 0.776 | 167.595 | **< 0.001** | 5.440 | **0.020** | 1.535 | 0.464 |

|  | **Bat Falcon** | | | | **Brown Falcon** | | | | **New Zealand Falcon** | | | | **Red-necked Falcon** | | | |
| --- | --- | --- | --- | --- | --- | --- | --- | --- | --- | --- | --- | --- | --- | --- | --- | --- |
| *Variable* | *Model* | *Estimate* | *t-value* | *p-value* | *Model* | *Estimate* | *t-value* | *p-value* | *Model* | *Estimate* | *t-value* | *p-value* | *Model* | *Estimate* | *t-value* | *p-value* |
| PC1 | LMM | -0.291 | -3.684 | **< 0.001** | LMM | 0.261 | 3.624 | **< 0.001** | LMM | -0.059 | -0.664 | 0.507 | LMM | 0.101 | 1.678 | 0.093 |
| PC2 | LMM | 0.150 | 2.481 | 0.013 | LMM | -0.002 | -0.029 | 0.977 | LMM | -0.046 | -0.561 | 0.575 | LMM | -0.039 | -0.675 | 0.500 |

**Table S5.** The relationships between the principal component axes and the average direct normal irradiation per species, including model type, the estimates per model, the test statistic, and the p-value
